# Supplementary material for: Intelligent Identification of Early Esophageal Cancer by Band-Selective Hyperspectral Imaging
Source: Cancers (Basel). 2022 Sep 1;14(17):4292. doi: 10.3390/cancers14174292 (PMC9454598; doi:10.3390/cancers14174292)
Supplement: Supplementary file 1 [file cancers-14-04292-s001.zip › cancers-1871917-supplementary.pdf]

cancers

Article

Intelligent Identification of Early Esophageal Cancer by Band-Selective Hyperspectral Imaging: Supplementary Material

S1. Single Shot Multi-Box Detector

In 2015, the Single Shot Multi-Box Detector (SSD) was proposed. It is constructed by a Convolutional Neural Network (CNN) and is a fast single-shot multi-category target detector. Figure S1 is the architecture diagram of SSD.

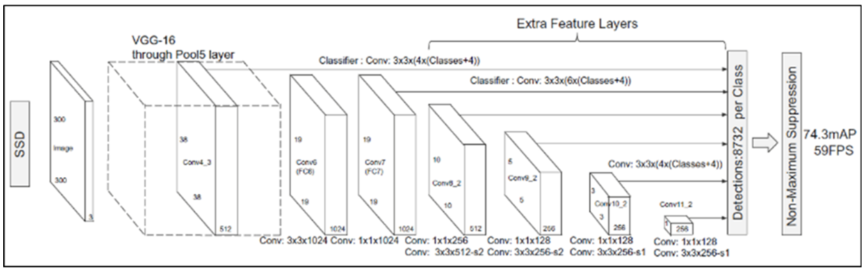

Figure S1. SSD Architecture Figure [1].

The SSD used in this study is a detection architecture based on the VGG-16-Atrous1 network. Vgg-16 is composed of 16 hidden layers with 13 convolutional layers and 3 fully connected layers. Figure S2 represents the architecture diagram of VGG16. SSD uses a feature extraction network, removes the two fully connected layers of fc6 and fc7, and adds a pyramidal feature hierarchy to detect feature maps of different sizes: large detection of small objects , small detection of large objects. In this way, the detection of small targets is strengthened, and the mechanism of anchors is used. Default boxes of a certain size are preset in advance, which can reduce the difficulty of training.

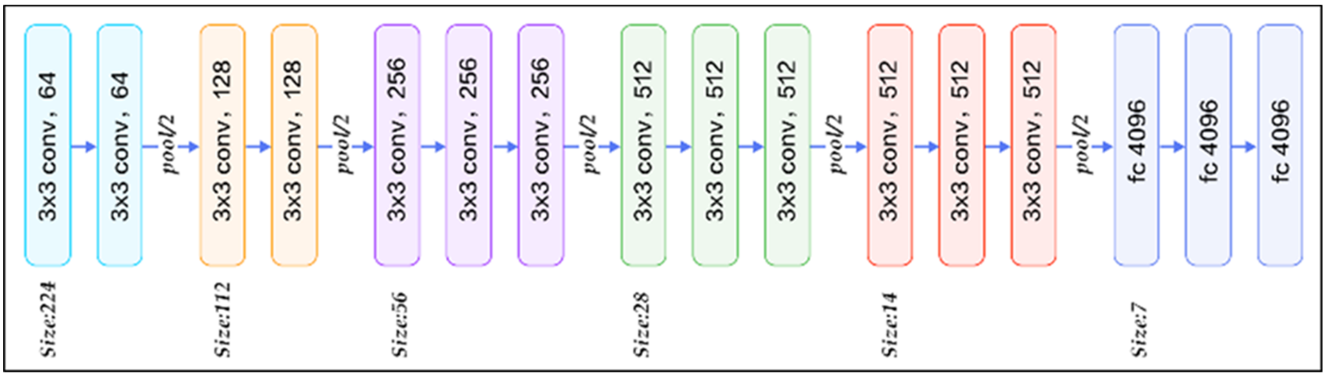

Figure S2 VGG16 Architecture Diagram. The red box is the VGG16 part; the yellow box is the pyramid structure

Using the pyramidal feature hierarchy as shown as a yellow box in Figure S2, has 6 layers of convolutional layers, so the size of the feature map is also reduced, and the detection scale is gradually reduced. The advantage is that it can be used for different sizes. The large feature map can be used to detect small objects, while the small feature map can be used to detect large objects, as shown in Figure S3.

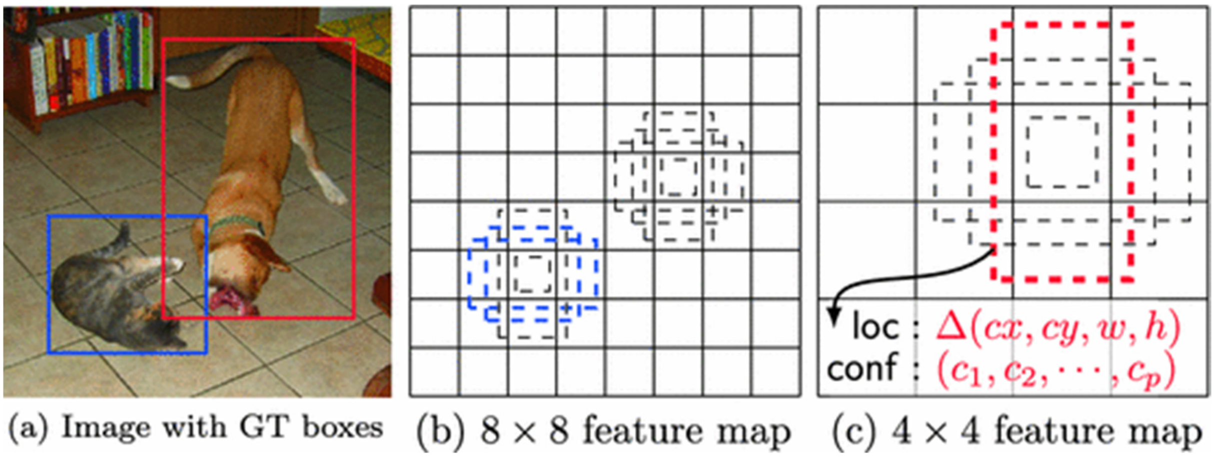

Figure S3. SSD Feature Map Scaling (a) shows the image with a real frame; (b) and (c) are two feature maps of different sizes, and finally, two kinds of results are output.

SSD will generate feature maps of different scales, and two 3x3 CNNs will be connected behind each feature map, one will output the location and the other will output the confidence, so as to obtain a series of prediction results. Figure S3 (c) shows the output position and confidence for each prediction box. In Figure S3, each small cell is called a feature map cell, and the preset default frame size and the relative position of the cell are fixed. The predicted frame is not an absolute position but is relative to the real frame offset. Therefore, combined with the previous two sections, the multiple feature maps generated by the pyramid structure will predict the object according to the preset default frame size, and generate a series of different types of position information and corresponding confidence.

S2. Matching strategy

In the training process, the method of selecting the default box corresponding to the real box is the Jaccard index, also known as intersection over union (IOU). First, the maximum IOU value will be matched first, and then a threshold will be set for the IOU, usually 0.5. The larger value is called the "positive sample", and the smaller value is called the "negative sample", as shown in Figure 4.

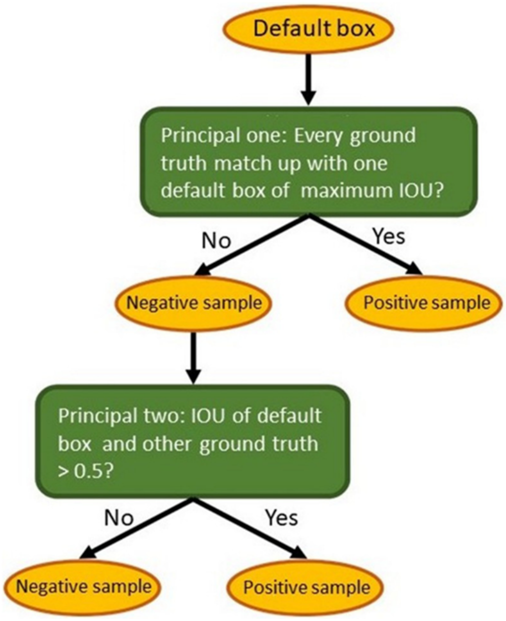

Figure S4 Default box matching diagram

In order to achieve the purpose of training, these default boxes are matched with the fact box, and two losses are used to calculate the error between the two, namely the confidence loss and the localization loss, the sum of which is the loss Function, as shown in Equation S1, and then train according to its size.

$$L(x,c,l,g) = \frac{1}{N}\left(L_{conf}(x,c) + \alpha L_{loc}(x,l,g)\right) \quad \text{(S1)}$$

Where,

$$L_{conf}(x,c) = -\sum_{i\in Pos}^{N} x_{ij}^{k} \, log\left(\hat{c}_i^{p}\right) - \sum_{i\in Neg} log(\hat{c}_i^{o})$$

$$L_{loc}(x,l,g) = \sum_{i\in Pos}^{N} \sum_{m\in\{cx,cy,w,h\}} x_{ij}^{k} \, \mathrm{smooth}_{L1}(l_i^m - \hat{g}_j^m)$$

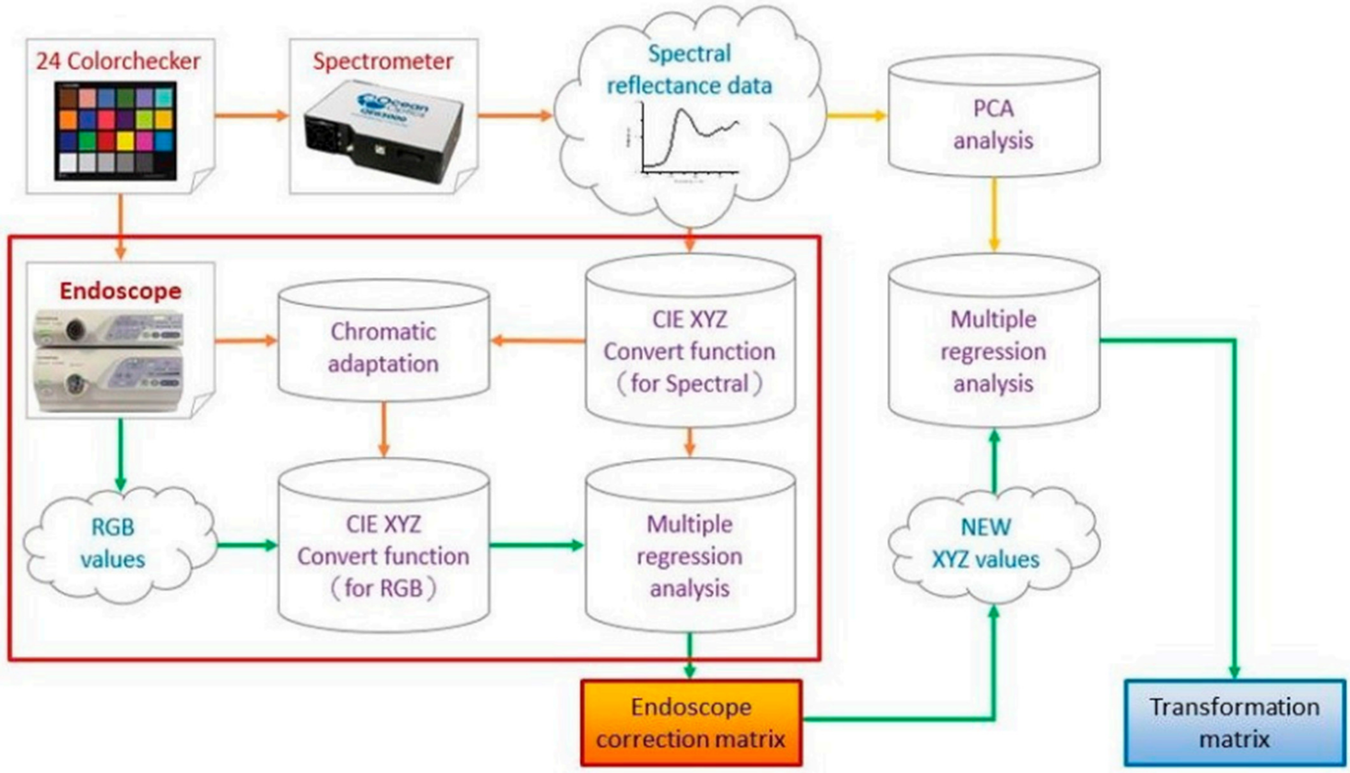

Figure S5. The HSI Algorithm

S3. Dataset Classification

Before the data enters the model, the data needs to be divided into a training dataset, a validation dataset, and a test dataset. Training set accounts for about 70% of the data set, which is put into the model so that the model can be trained and adjusted. A test set is created in order to test the results of training, and another completely independent data set is used to evaluate.

The number of WLI images is as follows: Normal 470; Dysplasia 156; SCC 219; The number of NBI images is as follows: Normal 425; Dysplasia 290; SCC 220; The images of WLI and NBI are divided according to the training set and test set. The number of WLI images in the training set is as follows: Normal 343; Dysplasia 102; SCC 156; The staging is as follows: Normal 775; Dysplasia 219; SCC 504; The number of NBI images by staging is as follows: Normal 345; Dysplasia 222; SCC 144; The number of WLI images in the test set is as follows: Normal 134; Dysplasia 51; SCC 59; The number of frames in the image is as follows: Normal 323; Dysplasia 76; SCC 174; The number of NBI images by staging is as follows: Normal 84; Dysplasia 74; SCC 66; The number of frames in the image is staging as follows: Normal 240; Dysplasia 195; SCC 120.

Table S1. Number of imaging data of esophageal cancer

	Stage	images	Total
WLI	Normal	470	845
	Dysplasia	156	
	SCC	219	
NBI	Normal	425	935
	Dysplasia	290	
	SCC	220	

Table S2. Classification of imaging data of esophageal cancer

		Stage	images	Total	boxes	Total
Train	WLI	Normal	343	601	775	1498
		Dysplasia	102		219	
		SCC	156		504	
	NBI	Normal	345	711	864	1797
		Dysplasia	222		531	
		SCC	144		402	
Test	WLI	Normal	134	244	323	573
		Dysplasia	51		76	
		SCC	59		174	
	NBI	Normal	84	224	240	555
		Dysplasia	74		195	
		SCC	66		120	

References

1. Simonyan, K. and A. Zisserman, Very deep convolutional networks for large-scale image recognition. arXiv preprint arXiv:1409.1556, 2014.

2. Liu, W., et al. Ssd: Single shot multibox detector. in European conference on computer vision. 2016. Springer.
